# Lyme beyond the season: late-autumn dissemination of infected ticks by southward migrating redwings (*Turdus iliacus*)

**DOI:** 10.1186/s13071-026-07673-x

**Published:** 2026-09-12

**Authors:** Dieter J. A. Heylen, Ignace Ledegen, Marc Grimon, Patrick Reteng, Judith M. Hübschen, Aleksandra Iwona Krawczyk

**Affiliations:** 1 Evolutionary Ecology Group, Department of Biology, University of Antwerp, Antwerp, Belgium; 2 Clinical and Applied Virology Group, Department of Infection and Immunity, Luxembourg Institute of Health, Esch-sur-Alzette, Luxembourg; 3 Certified Bird Ringer, Herentals, Belgium; 4 Certified Bird Ringer, Vorselaar, Belgium

**Keywords:** Climate change, Migration, Bird, Tick, *Borrelia*, Tick-borne encephalitis virus

## Abstract

**Background:**

Climate warming is altering the seasonal timing of many ecological interactions. Migratory birds typically time their movements using photoperiodic cues, whereas the activity of ectoparasites such as ticks is largely temperature dependent. This difference in environmental drivers may create climate-induced phenological mismatches that reshape host–parasite interactions.

**Methods:**

We investigated tick infestation in migratory thrushes during late-autumn migration in Belgium, a period historically assumed to fall largely outside the main activity season of *Ixodes ricinus*. In addition, we screened ticks collected from these birds for tick-borne pathogens. Both sampling years (2022 and 2024) showed unusually mild autumn conditions.

**Results:**

284 redwings (*Turdus iliacus*) were examined, revealing exceptionally high tick infestation rates (72.9%), the highest reported for this species in Europe. *Ixodes ricinus* predominated, and screening of 698 ticks showed that 12.9% carried *Borrelia* spp., including multiple avian- and mammalian-associated genospecies as well as *Borrelia miyamotoi*. Infected ticks were present on nearly one-third of birds. First calendar-year birds carried significantly higher tick loads than older individuals. Extrapolation to the European redwing population suggests that tens of millions of infected ticks may be transported southward each autumn.

**Conclusions:**

These findings indicate that late-autumn migration can coincide with substantial tick activity under mild climatic conditions, creating a previously overlooked seasonal window for parasite dispersal. Because migration timing is relatively stable whereas parasite activity is climate sensitive, continued warming is likely to strengthen this phenological overlap.

**Graphical abstract:**

**Figure:**
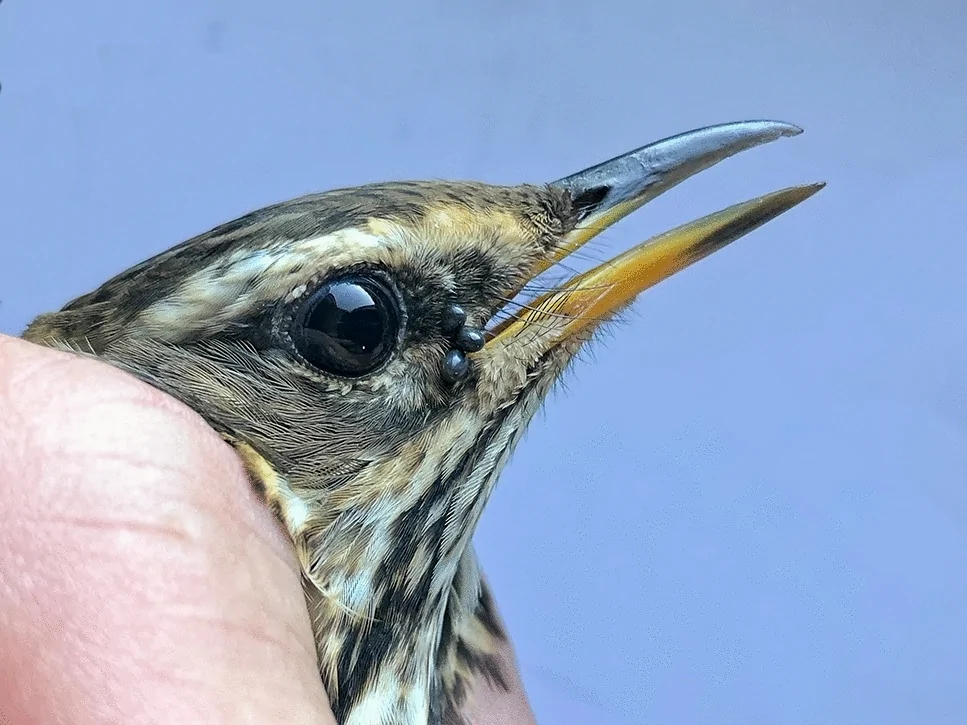

**Supplementary Information:**

The online version contains supplementary material available at https://doi.org/10.1186/s13071-026-07673-x.

## Background

Climate change has led to more favorable physiological conditions for ectothermic ectoparasites that spend parts of their lives off-host, especially in regions with cool climate conditions at high latitude and altitude. These climate shifts have resulted in prolonged host-searching seasons and increased both developmental and survival rates [1, 2]. Ticks of the holarctic region are no exception to this trend. *Ixodes ricinus* is Europe’s most important vector for zoonotic diseases [3, 4]. It typically shows a small autumn activity peak with individuals ‘questing’ for hosts [5], yet a growing number of studies demonstrate that with warmer autumn and winter conditions, ticks remain active during winter time [6, 7]. Moreover, its distribution has expanded northward [8], a trend paralleled by its North American sister species (*I. scapularis* and *I. pacificus*) [9].

Birds play a dual role in maintaining and dispersing ectoparasites and the pathogens these ectoparasites transmit [10, 11]. In Europe, many southward migrating birds that forage on the ground and lower vegetation strata of forests and pastures acquire tick infestations with immature developmental stages of *I. ricinus* [12, 13]. Typical *I. ricinus* tick habitats include the understory vegetation and leaf-layers of forests, recreational parks and gardens [4, 5]. After feeding for several consecutive days, engorged larval and nymphal ticks detach in the environment, where they molt to the next stage (nymphs and adults, respectively). During blood feeding on their migratory bird hosts, ticks may acquire avian pathogens—some of which are zoonotic [14, 15] and are transported across thousands of kilometers, spanning macro-geographical regions. Among the most commonly exposed birds are thrushes—members of the family Turdidae [16]. Many of them are ground-foraging, and given the thrushes’ large body size, hence skin surfaces on which ticks can feed, they have the potential to harbor large numbers of ticks [16]. Within Europe, all thrush species include migratory subpopulations, especially in northern regions [17–19].

Over recent decades, multiple studies have reported zoonotic pathogens isolated from bird-derived ticks, underscoring the epidemiological significance of avian hosts [12, 13, 20]. One of the most prominent is *Borrelia garinii*, a member of the *B. burgdorferi* sensu lato complex and the primary cause of Lyme neuroborreliosis in humans [21]. Much of the recent research on tick-borne pathogens and birds has focused on the discovery of new pathogenic agents that may expand the tick-borne pathogen community carried by birds. However, a deep understanding of the spatio-temporal variation in avian tick loads and pathogen transmission is challenging. Such assessments require longitudinal data on microhabitat use and host infectiousness, while tick activity itself—often modulated by climatic conditions at departure or stopover sites—adds another layer of complexity. These interacting processes operate on different environmental cues and temporal scales: in contrast to the temperature-driven host-searching activity of ticks [22], the timing of long-distance fall migration in many bird species is primarily governed by seasonal changes in daylength [23].

Here, to address the question to which extent southward migrating birds are contributing to the transmission of tick-borne pathogens during warm weather conditions beyond the typical tick season [5], we conducted intensive captures of thrushes during late autumn (November 2022 and 2024). Each year, millions of thrushes pass through Flanders (Belgium) situated at the crossroads of three major migratory flyways (North-East European, Scandinavian, and British). For both years, autumn temperatures were high (Fig. S1 and S2): the European average temperature over land (September–November) of 2022 and 2024 were the third and fifth highest on record for the season, 1.25 °C and 0.98 °C above the 1991–2020 average, respectively. Besides assessing the tick infestation levels, we aimed to evaluate whether birds are infectious at this late stage of migration. For that, we investigated whether, beyond the well-known avian *B. burgdorferi* s.l. genospecies (including *B. garinii*, *B. valaisiana* and *B. turdi*), the more elusive tick-borne encephalitis virus (TBEV) is transported and/or transmitted via the birds, as several of the flyways intersect with endemic foci of Lyme neuroborreliosis and tick-borne encephalitis.

## Methods

### Bird captures and tick sampling

Inspection and sampling of thrushes were conducted during the late-autumn migration periods of two years (November 2022 and November 2024) in the region between Antwerp and Herentals (Flanders, Belgium). Trained and experienced bird banders caught the birds using nets and lure birds. Bird capturing and handling was permitted by a ringing license of the Flemish Ministry, and an exemption for research on protected species. In November 2024, the head region of each bird was inspected for 2 min, and all visible ticks were removed with tweezers during an additional 2-min period. In 2022, birds were also inspected, but only their infestation status (infested or non-infested) was recorded. Age was recorded only for the 2024 captures and could be confidently assigned for 251 of the 284 *T*. iliacus individuals; birds for which age could not be determined with certainty were excluded from the age-specific analyses (see below). The day of capture, the ticks of 2024 were individually stored at − 25 °C. Within 7 days after capture, these frozen ticks were identified to species level using a stereomicroscope and standard identification keys [24–26], and stored at − 80 °C until molecular analysis. A small number of ticks (35 out of 733 ticks) were inadvertently damaged during removal from the birds or during morphological identification. These specimens were retained for the infestation analyses, but were excluded from molecular analyses because tissue damage could compromise pathogen detection.

### Nucleic acid extraction

Total nucleic acid was extracted using the QIAamp DNA Blood Mini Kit (Qiagen), as previously described [27]. According to the kit manual, this extraction kit co-purifies DNA and RNA. Ticks were individually disrupted and homogenized in in 300 µL of lysis buffer AL with 5 mm steel beads in the TissueLyser II (Qiagen). The mixture was centrifuged at 16,200×g for 4 min and then 200 µL of supernatant was transferred into a new tube. 200 µL of 99% ethanol was added to the supernatant, vortexed briefly, and then transferred to a silica column. Washing of the column was completed as described in the manufacturer’s instructions. Total nucleic acid was eluted in 100 μL of elution buffer. In each extraction run, a negative control (phosphate-buffered saline, PBS) and positive controls (PBS spiked with measles virus (RNA virus) and human adenovirus (DNA virus)) were included to monitor the extraction process. Extracted nucleic acids from the extraction controls were tested using TaqMan real-time assays targeting each virus [28, 29] to verify the nucleic acid recovery. In a separate validation study, the performance of the QIAamp DNA Blood Mini Kit for DNA and RNA recovery was compared with that of the QIAamp Viral RNA Mini Kit using tick lysates spiked with human adenovirus and measles virus. The two kits showed comparable performance, with a mean difference of less than one Ct value (manuscript in preparation).

### Detection of tick-borne pathogens

Detection of TBEV and *Borrelia* spp. was carried out using TaqMan real-time assays, as previously described [30, 31]. Reactions were performed with the Quantitect Probe RT-PCR Kit (Qiagen) or the Quantitect Probe PCR Kit (Qiagen) in a final volume of 20 µL, containing 4 µL of extracted nucleic acid. In each PCR run, negative and positive controls (Amplirun^®^ Borrelia DNA control or Amplirun^®^ TBEV RNA control) were included; water was used as negative control. TBEV detection was always done on freshly extracted nucleic acid to avoid RNA degradation due to freeze-thawing. Details of the target genes, PCR conditions, and primers and probes are provided in Table S1.

### *Borrelia burgdorferi* s.l. and *Borrelia miyamotoi* amplification

Samples positive for *Borrelia* spp. were subjected to nested PCRs to amplify fragments for sequencing for *B. burgdorferi* s.l. and *B. miyamotoi*. Details of the target genes, PCR conditions, and primer and probe concentrations are provided in Table S2. All PCR reactions were performed using Platinum™ Taq DNA Polymerase kit (Invitrogen), in a final volume of 25 µL, containing 5 µL of extracted nucleic acid. For the nested PCR, the amplicon of the first PCR was diluted 1:4 and 5 µL of the diluted amplicon were used as template. Amplicons were visualized using gel-electrophoresis on a 1.5% agarose gel stained with SYBR safe (Invitrogen). Single-band PCR products were purified using the QIAquick PCR Purification Kit, whereas multiple-bands PCR products were purified with QIAquick PCR and Gel Cleanup Kit following gel excision before Sanger sequencing.

### Statistical analysis

The data possessed a hierarchical structure, with observations on tick infestation loads (0–∞) and pathogen prevalence in ticks (proportion of infected ticks per bird, based on individual tick infection status [0/1]) recorded for individual birds nested within flocks. In order to obtain valid statistical inferences, the dependence structure of the data needed to be taken into account. For this purpose, generalized linear mixed models (GLMMs) were fitted to the data (see [32]) taking into account the statistical dependence of observations by adding random effects: ‘bird flock’, and ‘bird individual’ (nested within flock) when multiple responses were included for the same bird. The residuals for pathogen prevalence and tick infestation prevalence were assumed to follow a binomial distribution (logit link), whereas those for tick infestation loads were assumed to follow a negative binomial distribution (log link). Tick species, developmental stage, and bird age together with their interactions, and capture date were included as explanatory variables in the full model. Thus, the initial models included a limited set of explanatory variables that were selected a priori based on biological relevance and the objectives of the study. To obtain a parsimonious final model, a backward elimination procedure was applied, sequentially removing non-significant fixed effects while maintaining the hierarchical structure of the models. At each step, the fixed effect with the highest non-significant *P*-value (*P* > 0.05) was removed, after which the model was refitted. Model reduction continued until all remaining fixed effects were statistically significant (*P* < 0.05), while lower-order terms involved in significant interactions were retained [33]. The random effect of bird individual was retained in all models involving repeated observations from the same bird (i.e., multiple tick species and/or developmental stages recorded for the same bird) to account for within-individual dependence. An additional random effect of bird flock was initially included to account for potential clustering of birds within flocks, but was not retained in the final models because the estimated flock-level variance was consistently close to zero. The full GLMM for tick infestation loads did not converge. Therefore, these data were analyzed using a marginal modeling approach based on generalized estimating equations (GEE) (see [32]) accounting for repeated observations, with bird individual as the cluster variable, using an exchangeable working correlation structure and robust sandwich standard errors. For additional simple two-way comparisons, when counts were low (< 5), Fisher’s exact tests were applied instead of *χ*^*2*^ tests. All data management and statistical analyses were done in SAS v 9.4 (SAS Institute, Cary, North Carolina, USA).

## Results

### Tick infestation

**Tick prevalence**: In our study, the prevalence of ixodid tick infestations did not statistically differ between the 2 years, in any of the thrush species: *T. iliacus* (2022: 72.2%, *n* = 108 vs. 2024: 72.9%, *n* = 284; *χ*^*2*^ = 0.017; *df* = 1; *P* = 0.89), *T. pilaris* (0.0%, *n* = 5 vs. 7.1%, *n* = 56; *χ*^*2*^ = 0.38; *df* = 1;* P* = 0.53), *T. merula* (50%, *n* = 2 vs. 80%, *n* = 5; Exact Fisher* P* = 1) and *T. philomelos* (40.0%, *n* = 10 vs. 50.0%, *n* = 4; Exact Fisher* P* = 1).

Below we focus on *T. iliacus* caught during late autumn 2024 (*n* = 284). Species identification of the 733 isolated ticks (Table 1) revealed that the prevalence of *I. ricinus* (67.6%; 192 birds infested) was significantly higher than that of *I. frontalis* (10.6%; 30 birds infested; logit ∆ _*I.frontalis*-*I.ricinus*_: − 2.85 ± 0.24, *T*-value = − 12.03, *df* = 281,* P* < 0.001). Co-infestations with both species feeding together occurred in 5.3% of the birds.

**Table 1:** Infestation prevalence, load, and distributions over different developmental stages and tick species; data used from redwing (*Turdus iliacus*) captures in Belgium (November 2024)

	*Ixodes* spp. 733 ixodid ticks isolated from 207 infested birds	*Ixodes ricinus* 565 ticks isolated from 192 infested birds		*Ixodes frontalis* 168 ticks isolated from 30 infested birds	
284 birds caught	% infested (birds); Load ± Std	% infested (birds); Load± Std	Distribution Feeding status	% infested (birds); Load± Std	Distribution Feeding status
**All**	72.89(207); 3.69±9.90	67.61(192); 2.94±2.75		10.56(30); 5.60±14.74	
**Larva**	50(142); 2.55±9.62	39.44(112); 2.29±1.99		7.39(21); 7.43±17.42	
Unfed		8.45(24); 1.50±1.29	14.06% (36)	2.11(6); 1.00±0.00	3.85% (6)
Semi-engorged		28.52(81); 2.10±1.89	66.41% (170)	2.46(7); 1.57±1.51	7.05% (11)
Engorged		10.92(31); 1.61±1.38	19.53% (50)	4.23(12); 11.58±22.46	89.10% (139)
**Nymph**	53.87(153); 1.14±1.52	51.76(147); 2.10±1.50		3.52(10); 1.10±0.32	
Unfed		23.24(66); 1.58±0.77	33.66% (104)	0.70(2); 1.00±0.00	18.18% (2)
Semi-engorged		34.51(98); 1.78±1.29	56.31% (174)	1.06(3); 1.00±0.00	27.27% (3)
Engorged		9.86(28); 1.11±0.31	10.03% (31)	1.76(5); 1.20±0.45	54.55% (6)
**Adult**	0.35(1); 1	0(0)		0.35(1); 1	
Unfed		0 (0)	0% (0)		0% (0)
Semi-engorged		0 (0)	0% (0)	0.35(1); 1	100% (1)
Engorged		0 (0)	0% (0)		0% (0)

Co-infestations: 5.3% of the bird individuals infested with both tick species

Co-feeding larvae and nymphs: 23.6% and 0% of the birds for *I. ricinus* and *I. frontalis*, respectively

Tick prevalences showed a significant species x stage interaction (F_1, 1119_ = 8.99,* P* = 0.0028). *Ixodes ricinus*’ immature developmental stages were commonly found feeding on the birds (larval infestations: 39.4% of the birds; nymphal: 51.8%; logit ∆ _larva-nymph_: − 0.51 ± 0.17, *T*-value = − 3.07, *df* = 281.4,* P* = 0.0024) and were frequently observed co-feeding on the same host individuals (23.6% of the *I. ricinus*-infested birds). Co-feeding of different stages was not observed in *I. frontalis* in which the prevalence of larvae (7.4%) tended to be higher than that of nymphs (3.5%, logit ∆ _larva-nymph_: 0.78 ± 0.40, *T*-value = 1.96, *df* = 298.1,* P* = 0.05). No bird age differences in tick prevalences were detected in the subset of birds for which age could be confidently determined (logit ∆ _old – young_: − 0.45 ± 0.25, *T* = − 1.82, *df* = 279.1,* P* = 0.07).

Feeding adult females were only observed for *I. frontalis*. Besides the record in 2024 (Table 1), during the banding sessions in 2022, all eight adult female ticks belonged to *I. frontalis* (one each on eight different birds).

**Tick burden:** Tick loads showed a significant species x stage interaction (GEE; *χ*^*2*^ = 6.20; *df* = 1;* P* = 0.013), which was due to significantly lower *Ixodes frontalis* nymphal loads, compared to all other species x stage combinations (log ∆ _*Other—I.frontalis* nymph_: − 3.07 ± 0.35, Z-value = − 8.73,* P* < 0.001). When looking at the raw data, compared to average larval *I. ricinus* loads (2.29 ± 1.99 larvae per bird), *I. frontalis* larvae showed higher but more variable loads (7.43 ± 17.42 larvae per bird)—largely due to two individuals with loads of 80 (40 isolated) and 134 (74 isolated) larvae each. First-calendar-year individuals showed higher tick loads than older birds (GEE; log ∆ _old – young_: − 0.73 ± 0.26, Z = − 2.80, *df* = 200,* P* = 0.005).

**Tick feeding status:** Overall, while for *I. ricinus* the most commonly found feeding status was semi-engorged (66.4% of the 256 larvae and 56.3% of the 309 nymphs), *I. frontalis* ticks were more often found fully engorged (89.1% of the 156 larvae and 54.6% of the 11 nymphs).

### TBEV and *Borrelia* infections

A total of 698 ticks collected from 201 T*. iliacus* individuals were screened for TBEV and *Borrelia* spp. None of the ticks tested positive for TBEV, whereas 12.9% (*n* = 90) tested positive for *Borrelia* spp. (Table 2). All positive extraction controls tested positive for the respective viruses and Ct values were within the expected range. At the host level, the proportion of *I. ricinus*-infested birds carrying *Borrelia*-infected ticks (29.6%, *n* = 189 birds) was higher than that of *I. frontalis*-infested birds (20.0%, *n* = 30 birds), but the difference was not statistically significant (χ^2^ = 1.18; *df* = 1;* P* = 0.28). Among *I. ricinus*-infested birds, 23.8% carried one or more infected larvae and 31.7% carried one or more infected nymphs. In *I. frontalis*-infested birds, these prevalences were lower (15.3% for infected larvae and 10.0% for infected nymphs).

**Table 2:** *Borrelia* spp. prevalence in screened ticks collected from infested redwings (*Turdus iliacus*) in Belgium (November 2024)

		Total	Larva	Nymph	Adult
*Ixodes* sp.	No of screened ticks (infested birds)	698 (201)	388 (123)	309 (150)	1 (1)
	Prevalence *Borrelia*-infected ticks (infected birds) %	12.9 (29.4)	8.8 (17.9)	18.1 (31.3)	0.0 (0.0)
*Ixodes ricinus*	No of screened ticks (infested birds)	541 (189)	243 (111)	298 (145)	
	Prevalence *Borrelia*-infected ticks (infected birds) %	14.0 (29.6)	8.6 (23.8)	18.5 (31.7)	
*Ixodes frontalis*	No of screened ticks (infested birds)	157 (30)	145 (21)	11 (10)	1 (1)
	Prevalence *Borrelia*-infected ticks (infected birds) %	8.9 (20.0)	9.0 (15.3)	9.1 (10.0)	0.0 (0.0)

Interpretation of the prevalence in birds for a particular tick species x stage combination: the proportion of birds carrying at least one infected tick in the pool of birds infested with that combination

At the tick level, *Borrelia* spp. prevalence in *I. ricinus* (14.0%, 76 out of 541 ticks) was higher than in *I. frontalis* (8.9%; 14 out of 157 ticks), but this difference was not statistically significant (logit ∆ _*I. frontalis-ricinus*_: − 0.08 ± 0.34, *T*-value = − 0.23, *df* = 694,* P* = 0.81) taking into account the correlation within bird individuals (σ^2^: 1.01 ± 0.05; Z = 181.63;* P* < 0.001) and controlling for tick instar (logit ∆ _larva-nymph_: − 0.81 ± 0.26, *T*-value = − 3.13, *df* = 694,* P* = 0.0018). Among birds included in the pathogen analyses for which age could be determined (*n* = 182), the proportion of *Borrelia*-infected ticks did not differ significantly between age classes (logit ∆ _old – young_: − 0.26 ± 0.43, *T* = − 0.61, *df* = 641,* P* = 0.54).

### *Borrelia* genospecies and genotype distribution

In total, six known *B. burgdorferi* s.l. genospecies were detected in the ticks collected from *T. iliacus* (distributed across 75 ticks), along with the relapsing fever spirochete *B. miyamotoi* (*n* = 10 ticks). The obtained *B. miyamotoi glpQ* sequences were identical to those from strains NL-IR-1, ZStrull14-9, and CZ-F190E, which were isolated from *I. ricinus* in the Netherlands, Germany, and Czech Republic, respectively, indicating the European clade [34]. The highest genospecies diversity occurred in *I. ricinus* nymphs, which harbored both avian-associated genospecies (*B. turdi*, *B. garinii*, *B. valaisiana*) and mammalian-associated genospecies (*B. burgdorferi* sensu stricto, *B. spielmanii*, *B. afzelii*). In *I. frontalis* ticks, only *B. garinii* and *B. valaisiana* were detected (Table 3).

**Table 3:** Distribution of *Borrelia* genospecies in *Ixodes ricinus* and *I. frontalis* ticks collected from redwings (*Turdus iliacus*) in Belgium (November 2024)

	*Ixodes ricinus*				*Ixodes frontalis*			
		Larva	Nymph	Adult	Total	Larva	Nymph	Adult
*B. afzelii*	3 (4.5%)	0	3	-	0 (0%)	0	0	-
*B. burgdorferi* s.s.*	1 (1.5%)	0	1	-	0 (0%)	0	0	-
*B. garinii*	38 (56.7%)	14	24	-	5 (62.5%)	4	1	-
*B. miyamotoi*	10 (14.9%)	3	7	-	0 (0%)	0	0	-
*B. spielmanii*	1 (1.5%)	0	1	-	0 (0%)	0	0	-
*B. turdi*	1 (1.5%)	0	1	-	0 (0%)	0	0	-
*B. valaisiana*	8 (11.9%)	1	7	-	3 (37.5%)	3	0	-
*B. afzelii* and* B. miyamotoi*	1 (1.5%)	0	1	-	0 (0%)	0	0	-
*B. garinii* and* B. valaisiana*	4 (6%)	1	3	-	0 (0%)	0	0	-
Total sequenced	67	19	48		8	7	1	

*sequence closely resembles that of *B. burgdorferi s.s. finlandensis* [35], although not yet officially recognized [36]

Amplicon analyses of 333 bp-long *flaB* sequences further revealed 17 potential genotypes within these genospecies. Three of the four genotypes found in *I. frontalis* were also present in the larger collection of *I. ricinus* ticks. Among birds with co-infestations, one individual carried larvae of both tick species infected with the same *B. garinii* genotype, and another carried ticks infected with *B. miyamotoi* (*I. ricinus*) and *B. garinii* (*I. frontalis*).

Co-infections of *B. garinii* and *B. valaisiana* were observed in four ticks from four individual birds. One bird carried an *I. ricinus* nymph co-infected with *B. afzelii* and *B. miyamotoi*. Nine birds carried ticks infected with more than one genospecies across separate ticks—seven with *B. garinii* and *B. miyamotoi*, and two with *B. garinii* and *B. valaisiana*. In addition, five birds carried ticks infected with more than one *B. garinii* genotype. Notably, none of the ticks infected with mammalian-associated genospecies (*B. afzelii*, *B. burgdorferi* s.s., *B. spielmanii*) were observed co-feeding with other ticks carrying the same genospecies. All unique sequences of *B. burgdorferi* s.l. and *B. miyamotoi* generated in this study have been submitted to GenBank under accession numbers PX741403–PX741426.

### Variation between bird flocks and capture dates

Variation between bird flocks did not significantly differ from zero, neither in the overall ixodid infestation loads and prevalences, nor at the tick species level. During one capture date (November 5, 2024), significantly higher *I. ricinus* prevalences (logit: 1.26 ± 0.32, *T*-value = 3.98, *df* = 281.8,* P* < 0.001) and loads (log: 0.91 ± 0.20, *T*-value = 4.57, *df* = 200.8,* P* < 0.001) were observed than during the other capture events. Variation between bird flocks in their contribution to *Borrelia* transmission (in terms of infected ticks and the prevalence of birds carrying infected ticks) did not differ significantly from zero for any tick species or instar.

Sample sizes were too small to assess differences in the composition of avian genotypes among bird flocks (range of genotype × bird combinations per flock: 0–4). However, when examining capture dates (range genotype × bird combinations: 1–11), the composition differed significantly among the four dates with at least eight genotype × bird combinations (November 2 to 5, 2024; *n* = 38; Fisher’s exact test, *P* = 0.01).

## Discussion

Our results show that late-autumn migration can coincide with substantial tick infestation during unusually warm autumn conditions, revealing a previously underappreciated seasonal window for parasite dispersal [5]. High tick prevalences were registered in *T. iliacus*, which was the most abundant bird species migrating through at this time of the year. A literature overview (Table S4) indicates that the infestation levels are among the highest recorded in the past 70 years. The entire migrating European population of *T. iliacus* during autumn counts 16–28 million mature individuals and 120–210 million first year birds [18]. Assuming these birds experience similar tick exposure and *Borrelia* spp. infections as observed in our study (0.22 infected larva/bird and 0.21 infected nymph/bird; based on tick loads and *Borrelia* spp. prevalence of Table 1 and 2), we project that in autumn 2024, this single bird species may have transported nearly 100 million infected ticks to southern West-Palearctic areas. Given that our late-autumn estimates of tick infestation levels would be on the lowest boundary—due to *I. ricinus*’ phenology [5]—this projection may be even an underestimation. However, this projection should be interpreted cautiously, as it is based on observations from a single Belgian migration stopover and assumes that infestation and infection levels are broadly representative of migrating redwings throughout Europe.

Consistent with the historical data (Table S4), infestations of *T. pilaris* were much rarer. Furthermore, *T. merula*, which mainly migrates at night, and *T. philomelos* were not covered by sufficient numbers of birds to obtain reliable epidemiological estimates, but the two species together (11 infested out of 21 birds) provided more infested birds than all *T. pilaris* together (4 out of 61 birds).

To explain tick infestation patterns in the bird communities, the spatio-temporal overlap between the ticks and hosts is the primary determinant [10, 13], although behavioral resistance (e.g., grooming and preening) [37] and feather properties likely influence infestation successes as well. Thrushes spend considerable time above the lower vegetation strata (e.g., when roosting, finding shelter, or foraging higher in bushes and trees) where they are out of reach of questing ticks [5, 38]; however, main dietary food items are to be found close to the ground (e.g., berries in shrubbery, or invertebrates [18]) drawing them downward. *Turdus iliacus*, *T. merula* and *T. philomelos* are more frequently exposed to the generalist tick *I. ricinus*, because they forage in prime tick habitats such as wooded areas, forest edges and parks. In contrast, the open grasslands and meadows preferred by *T. pilaris* are in general less suitable for ticks, as these habitat types experience more direct sunlight and summer drought, which reduce tick survival; for example immature stages of *I. ricinus* require relative humidity levels of at least 80% [39]. Unlike other thrushes, *T. pilaris* rarely scratches in leaf litter [40] which forms a buffered microhabitat known to increase *I. ricinus* survival rates [5], reducing their infestation risk. Beyond habitat use, host age appears to shape infestation loads as well. Among *T. iliacus* that could be confidently aged, first-calendar-year birds carried higher tick loads than adults—a pattern consistent with either greater exposure in young, inexperienced birds or with age-related acquired resistance, though our data cannot distinguish between these mechanisms.

**Fig. 1:**
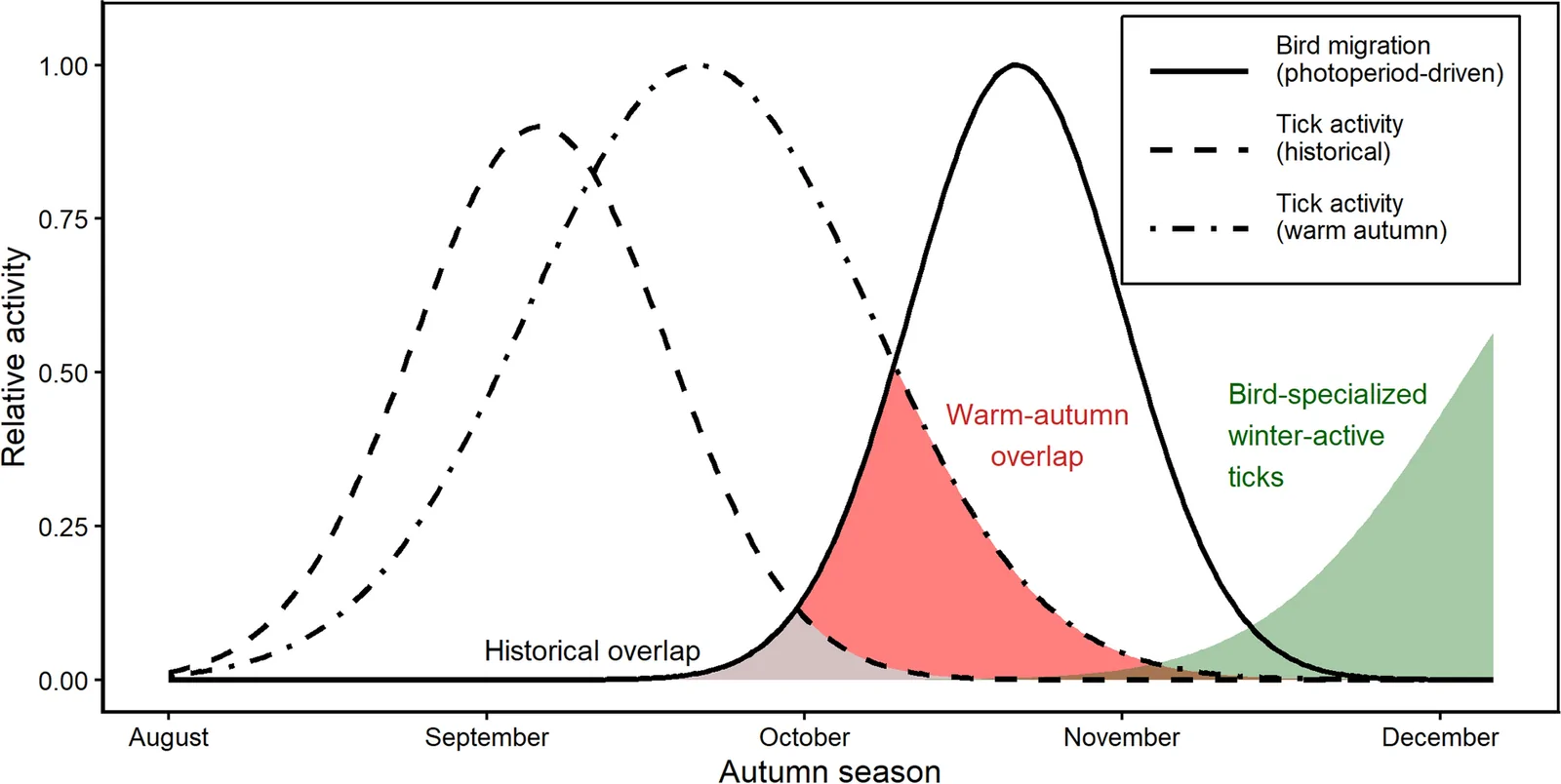
Hypothetical model illustrating how warmer autumn conditions may extend tick activity and increase phenological overlap with migratory thrushes. Migration timing in many species is largely photoperiod-driven and therefore relatively stable among years, whereas the ectothermic *Ixodes ricinus* tick’s questing activity is largely temperature-dependent. Warmer autumn conditions in the north may extend *I. ricinus* activity into late autumn, increasing phenological overlap and creating a larger seasonal window for long-distance dispersal of ticks and tick-borne pathogens. Co-infestations with winter-active bird-specialized ticks (e.g., *I. frontalis*) may further increase transmission events

The spatio-temporal overlap with *I. ricinus* observed in our study may reflect an increasingly likely process at the macro-spatial scale: Many migratory birds time their movements primarily in response to photoperiod, whereas tick activity is strongly temperature dependent, potentially creating phenological decoupling between host migration and parasite activity. Warmer autumn conditions— as observed during the past years in Europe (Fig. S1 and S2)—are expected to increase the temporal overlap between actively questing *I. ricinus* ticks on migrating birds, potentially resulting in high infestation levels (Fig. 1). Co-infestations with winter-active bird-specialized ticks, e.g., *I. frontalis*, may further increase transmission events (Table 1). *Ixodes frontalis* is typically found in relatively rare locations associated with communal avian roosting sites [41–44], explaining the low number of infestations in the migratory thrushes. We hypothesize that the high loads of fully engorged larvae observed in a few *T. iliacus* individuals (Table 1) reflect a single encounter with a concentrated batch of larvae at one of these localized roosting sites.

The complete absence of adult *I. ricinus* on the examined migratory thrushes is consistent with the ecology of this species. Whereas larvae and nymphs frequently parasitize birds, adult *I. ricinus* primarily exploit medium- to large-sized terrestrial mammals [4]. Adult females require comparatively large blood meals, and although mating can occur both on and off the host, copulation commonly occurs on the host [45]. Larger mammalian hosts may therefore provide more favorable conditions for adult reproduction than small passerines because their greater body surface area increases the likelihood that individual hosts simultaneously harbor multiple adult ticks, thereby increasing opportunities for encounters between males and feeding females. In contrast, the regular occurrence of adult *I. frontalis* is consistent with the stronger avian specialization of this species.

Overall, the *Borrelia* spp. prevalence in the isolated ticks was higher than in European questing *I. ricinus* ticks, especially when focusing on the avian genospecies (*B. garinii* and *B. valaisiana*) of *B. burgdorferi* s.l. (bird vs. vegetation larvae: 8.6% vs. 0%; bird vs. vegetation nymphs: 14% vs. 2.7% [46]). Therefore, we conclude that in urbanized areas like Flanders (Belgium) *T. iliacus* may contribute to the introduction of (novel) pathogenic agents and contribute to the endemic cycles at stopover sites. This is consistent with recent multilocus sequence typing work from Central Europe, where thrushes—particularly the Eurasian blackbird (*Turdus merula*)—were identified as key local reservoirs harboring a genetically diverse pool of *B. garinii* sequence types, including birds carrying multiple co-infecting genotypes simultaneously, underscoring the role of avian hosts not only in transmitting but also in generating and dispersing the strain diversity of this spirochete [47]. Because *B. burgdorferi* s.l. spirochetes are not transmitted trans-ovarially (i.e., mother-to-larva transmission) [48], their presence in several feeding larvae indicates that they were acquired via the host, either by systemic infections or by the less efficient co-feeding transmission [13, 49]. In the birds, in addition to recent infections via *Borrelia*-infected nymphs, latent infections acquired months before capture may become re-activated due to migratory restlessness. This has been shown in the lab with experimentally infected juvenile *T. iliacus* where *Borrelia* spirochetes re-appeared in the birds’ blood [15]. Infection prevalences were lower than in previous studies reporting on *T. iliacus* (Table S5). Several explanations can be put forward. First, thrushes do not necessarily remain infectious after a natural infection, and renewed exposure may be required to regain infectiousness [14]. It is therefore possible that birds in our study experienced fewer recent exposures in the weeks prior to capture compared with birds in earlier studies. Demographic differences may also contribute: first-calendar-year birds are known to migrate later in autumn than adults (M. Grimon and I. Ledegen, personal communication), but—because of their young age—have been less exposed to ticks and tick-borne pathogens than adult birds. Finally, from an evolutionary perspective, if proliferation is costly for the spirochetes themselves (e.g., through increased host immune responses, or negative effects on host survival), selection may favor low host infectiousness during periods when transmission opportunities are generally low, such as late autumn when tick densities should be low [5].

Although the two tick species (*I. ricinus* and *I. frontalis*) have different off-host ecologies [24, 43], they turn out to share bird individuals, and also share several of the studied pathogens. Significant numbers of *Borrelia* infections (including *B. garinii* and *B. valaisiana*) in the bird-specialized *I. frontalis* suggest it may act as a competent *Borrelia* vector, similarly to *I. ricinus*. One should mention that the presence of the bacteria in the ticks during feeding does not necessarily mean that the tick will effectively transmit the pathogens through salivation when feeding on the next host. Many correlative studies have shown that ticks are *Borrelia* carriers, but proving the vector competence of the ticks requires experimental investigations [50]. Such vector-competence experiments using a system with the blackbird *T. merula* and two *Borrelia* genospecies proved that *I. frontalis* is able to transmit *B. turdi* but not *B. valaisiana* [51]. Whether the same outcomes hold for closely related thrush species (e.g., *T. iliacus*, *T. philomelos* and *T. pilaris*) and/or different genospecies, like *B. garinii* [52] remains to be investigated.

Also for the more sporadically observed pathogens that have been linked to infectious mammals (*B. burgdorferi* s.s., *B. afzelii*, *B. spielmanii*) and were detected here in bird-derived nymphs, experiments are needed to investigate whether viable bacteria have survived the bird immune response [53] or whether the detected *Borrelia* DNA originates from non-viable bacteria acquired by larvae during their first blood meal on an infectious mammal. One of the *B. burgdorferi* s.s. sequences was closely related to *B. burgdorferi s.s. finlandensis*, which was described from Finland and proposed as a new *Borrelia* genospecies [35], although not yet officially recognized [36]. Whether birds play an indirect role by just dispersing ticks with viable mammal-associated spirochetes, rather than acting as competent reservoirs capable of bird-to-tick transmission, remains to be elucidated.

Regarding the relapsing fever spirochete *B. miyamotoi*, studies in the USA and Europe have implicated small rodents as the reservoir hosts [54, 55]. In contrast to *B. burgdorferi* s.l., *B. miyamotoi* spirochetes can also be transmitted vertically, i.e., from infected mother tick to her larvae [54, 56]. Therefore, *B. miyamotoi* in larval or nymphal ticks feeding on birds, observed here and in a few other studies [57–59], may reflect trans-ovarial infections or pathogen acquisition during feeding prior to attachment to the bird. Our observations do not prove that the bird was an infectious reservoir, for which xenodiagnosis and controlled challenge studies are needed.

Although many *T. iliacus* populations passing through Northwestern Europe originate from areas endemic for TBEV [40, 60], none of the birds in our study carried ticks, in which TBEV RNA was detected. In TBEV epidemiology, contributions and role of abundant groups of vertebrates (e.g., birds) are still much under debate [61]. Birds are notorious for actively contributing to the transmission of flaviviruses like West-Nile and Usutu viruses to which also TBEV belongs. However, from our study and others ([62] and references herein), viremia in *T. iliacus* during autumn migration appears unlikely. Nevertheless, because TBEV typically occurs at low prevalence and in highly localized foci, larger numbers of ticks and hosts would be required to conclusively exclude a role for migrating thrushes.

This study has several limitations that should be considered when interpreting the results. First, our observations are based on two autumn migration seasons, both characterized by unusually warm weather, and were obtained from a single migration stopover in Belgium. Longer-term monitoring across multiple years and regions will be required to determine whether the proposed climate-driven mechanism consistently explains late-autumn tick infestation patterns. Furthermore, although *Borrelia* spp. were detected in feeding ticks, the birds themselves were not directly tested for infection, precluding direct assessment of their infection status. Therefore, the presence of infected larvae provides strong evidence that birds can contribute to pathogen transmission, although direct testing of the birds would be required to confirm systemic infection.

## Conclusions

In conclusion, this study highlights that even during late autumn, large numbers of ticks—partly carrying zoonotic pathogens—are transported southward across Europe by redwings (*T. iliacus*). At the northern edge of *I. ricinus*’ range, ongoing climatic warming is expected to increase habitat suitability and milder autumn conditions may prolong tick activity, thereby increasing opportunities for tick dispersal and pathogen transmission via late-autumn migratory thrushes. Because successfully fed ticks are a prerequisite for (re-)exposure to tick-borne pathogens, fundamental ecological bird research into exposure risk factors remains essential. In addition, although temporal variation in susceptibility and infectiousness has hardly been studied at the level of host individuals, it is fundamental for our understanding of the circulation of tick-borne pathogens and zoonotic diseases in general.

## Supplementary Information

**Additional file 1**: **Table S1**. List of primers and probes used for pathogen detection. **Table S2**. List of primers used for sequencing. **Table S3**. GenBank accession numbers of flaB sequences used for Borrelia burgdorferi s.l. genospecies identification. **Table S4**. The tick-infested proportion of migratory birds – from literature. **Table S5**. *Borrelia* spp. prevalence in screened ticks collected from the five bird species in Belgium (November 2024) in comparison with estimates from literature data. **Fig. S1**. European-mean surface air temperature anomalies. **Fig. S2**. Spatial variation in surface air temperature anomalies.

## Data Availability

The datasets generated and/or analysed during the current study are all included in the main text.

## Abbreviations

TBEV: Tick-borne encephalitis virus
DNA: Deoxyribonucleic acid
GLMM: Generalized linear mixed model
RT-PCR: Real-time polymerase chain reaction
RNA: Ribonucleic acid
