# Supplementary material for: Lyme beyond the season: late-autumn dissemination of infected ticks by southward migrating redwings (Turdus iliacus)
Source: Parasit Vectors. 2026 Sep 12;19:397. doi: 10.1186/s13071-026-07673-x (PMC13576330; doi:10.1186/s13071-026-07673-x)
Supplement: Supplementary file 1 [file 13071_2026_7673_MOESM1_ESM.docx]

**Table S1.** List of primers and probes used for pathogen detection.

| Target pathogen | Target gene | Name | Sequence 5' to 3' | Final concentration (nM) | Annealing temp | Remarks | Ref |
| --- | --- | --- | --- | --- | --- | --- | --- |
| TBEV | 3'-UTR | F-TBE 1 | GGGCGGTTCTTGTTCTCC | 25 | 60°C | RT step: 50°C for 30 minutes | [1] |
|  |  | R-TBE 1 | ACACATCACCTCCTTGTCAGACT | 150 |  |  |  |
|  |  | TBE-Probe-WT | **FAM**-TGAGCCACCATCACCCAGACACA-**BHQ1** | 100 |  |  |  |
| *Borrelia* spp*.* | 16S rRNA | 16SBOR_F | GGTCAAGACTGACGCTGAGTCA | 400 | 60°C |  | [2] |
|  |  | 16SBOR_R | GGCGGCACACTTAACACGTTAG | 400 |  |  |  |
|  |  | 16SBOR_P | **FAM**-TCTACGCTGTAAACGATGCACACTTGGTG-**BHQ1** | 200 |  |  |  |

**Table S2.** List of primers used for sequencing.

| Target pathogen | Target gene | Direction | Name | Sequence 5' to 3' | Amplicon size | Final concentration (nM) | Annealing temp | Remarks | Ref |
| --- | --- | --- | --- | --- | --- | --- | --- | --- | --- |
| *Borrelia burgdorferi* s.l. | *flaB* | F1 | Outer1_BoR | AARGAATTGGCAGTTCAATC | ~490 | 400 | 51°C | Nested PCR | [3] |
|  |  | R1 | Outer2_BoR | GCATTTTCWATTTTAGCAAGTGATG |  | 400 |  |  |  |
|  |  | F2 | Inner1_BoR | ACATATTCAGATGCAGACAGAGGTTCTA | ~360 | 400 | 53°C |  |  |
|  |  | R2 | Inner2_BoR | GAAGGTGCTGTAGCAGGTGCTGGCTGT |  | 400 |  |  |  |
| *Borrelia miyamotoi* | *glpQ* | F1 | Q1 | CACCATTGATCATAGCTCACAG | 599 | 400 | 55°C | Nested PCR | [4] |
|  |  | R1 | Q2 | CTGTTGGTGCTTCATTCCAGTC |  | 400 |  |  |  |
|  |  | F2 | Q3 | GCTAGTGGGTATCTTCCAGAAC | 453 | 400 | 55°C |  |  |
|  |  | R2 | Q4 | CTTGTTGTTTATGCCAGAAGGGT |  | 400 |  |  |  |

**Table S3.** GenBank accession numbers of *flaB* sequences used for *Borrelia burgdorferi* s.l. genospecies identification.

| Genospecies | GenBank Accession number |
| --- | --- |
| *Borrelia afzelii* | GQ918147, PQ473548, ON755959 |
| *Borrelia bavariensis* | MN958342, MW963152 |
| *Borrelia bissettii* | KC003469 |
| *Borrelia burgdorferi* s.s. | MW963153, DQ016620, HM345911, MF150046, CP124070 |
| *Borrelia garinii* | ON755926, PQ473578, MF150065 |
| *Borrelia lusitaniae* | MW791417, DQ016623, PQ473601 |
| *Borrelia spielmanii* | MF150076, PV173342 |
| *Borrelia turdi* | MT235241, ON310824 |
| *Borrelia valaisiana* | MW963154, MT358278, DQ650330 |

**Table S4.** The tick-infested proportion of migratory birds for those year x season x species combinations for which at least 20 individuals were caught.

| **Species** | **Country** | **Author** | **Moment** | **% birds infested** | **Theoretical tick activity** |
| --- | --- | --- | --- | --- | --- |
| ***Turdus iliacus*** |  |  |  |  |  |
| 1&2 out of 8 | **Belgium** | **Current study** | **11.01-11.15 of 2024** | **73 (207/284)** | **Low** |
|  | **Belgium** | **Current study** | **11.01-11.15 of 2022** | **72.2 (78/108)** | **Low** |
|  | Poland | [5] | Spring 1981 | 40 (8/20) | High |
|  | Sweden and Denmark | [6] | Spring 1991 | 38 (15/40) | High |
|  | Sweden | [7] | 03.17 -05.30, and 07.07 - 11.13 of 2001 | 18 (9/51) | (*) |
|  | Sweden and Denmark | [6] | Autumn 1991 | 6 (23/417) | Medium |
| ***Turdus pilaris*** |  |  |  |  |  |
| 3 out of 6 | Sweden | [7] | 03.17 -05.30, and 07.07 - 11.13 of 2001 | 13 (3/23) | (*) |
|  | Norway | [8] | Spring 2008 | 10 (3/29) | High |
|  | **Belgium** | **Current study** | **11.01-11.15 of 2024** | **7.1 (4/56)** | **Low** |
|  | Norway | [8] | Autumn 2008 | 4 (2/48) | Medium |
|  | Sweden and Denmark | [6] | Spring 1991 | 4 (4/109) | High |
| Note:  Studies reporting pooled spring and autumn data are indicated separately (*). For *T. iliacus* and *T. pilaris* (> 20 individuals each), the data resulting from bird captures in Belgium (November 2022 and 2024) are situated within the top five highest known proportions of the past 55 years. The literature review is based on the updated database on infection prevalence of *B. burgdorferi* s.l. and tick burdens of hosts provided in [9]. Theoretical host-searching activity (‘low’ to ‘high’) of *Ixodes ricinus*, the main tick vector in Europe, is based on [7]. | | | | | |

**Table S5.** *Borrelia* spp. prevalence in screened ticks collected from the five bird species in Belgium (November 2024) in comparison with estimates from literature data.

|  | **% positive larvae**  **(ticks screened; number of studies)** | | **% positive nymphs**  **(ticks screened; number of studies)** | |
| --- | --- | --- | --- | --- |
| **Host species** | Literature data | Current study | Literature data | Current study |
| *Turdus iliacus* | 25.9 ± 5 (68;7) | 8.6 ± 1.8 (243;1) | 24.1 ± 2.7 (240;8) | 18.5 ± 2.2 (298;1) |
| *Turdus pilaris* | 33.3 ± 27.2 (3;1) | 0 ± 0 (1;1) | 11.3 ± 8.3 (19;3) | 0 ± 0 (2;1) |
| *Turdus merula* | 14.5 ± 1.1 (770;19) | 16.7 ± 15.2 (6;1) | 20.8 ± 0.8 (2138;24) | 33.3 ± 19.2 (6;1) |
| *Turdus philomelos* | 18.5 ± 1.6 (408;14) | 0 ± 0 (1;1) | 15.6 ± 1.3 (633;16) | 50 ± 35.4 (2;1) |
| Note:  Standard errors were calculated based on the binomial distribution assumption for prevalence proportions, and overall estimates were obtained through inverse-variance weighted meta-analysis. The literature review is based on the updated database on infection prevalence of *B. burgdorferi* s.l. and tick burdens of hosts provided in [9].Studies used: [6, 7, 8, 10, 11, 12, 13, 14, 15, 16, 17, 18, 19, 20, 21, 22, 23, 24, 25, 26, 27, 28, 29] | | | | |

Table S5 summarizes *Borrelia* spp. prevalence among ticks collected from thrush species. The proportion of positive *I. ricinus* larvae in this study (8.6%, n = 243) was significantly lower than the mean reported across seven studies (25.9 ± 5.0%; logit = −1.42 ± 0.35; T = −3.99, df = 6, P = 0.0072; total larvae n = 68). In contrast, the proportion of positive *I. ricinus* nymphs (18.5%) did not differ significantly from previous observations (24.1 ± 2.7%; logit = −0.47 ± 0.21; T = −2.27, df = 7, P = 0.06; total nymphs n = 240).

**Fig. S1.** European-mean surface air temperature anomalies relative to 1991-2020 for October and November, each (ERA5 data obtained via the Copernicus Climate Change Service: <https://climate.copernicus.eu/surface-air-temperature-maps>).

**Fig. S2.** Spatial variation in surface air temperature anomalies with reference period 1991-2020 for October and November of the years 2022 and 2024 (maps based on ERA5 data, and analyzed by the Copernicus Climate Change Service: https://climate.copernicus.eu/surface-air-temperature-maps). Dotted lines border the breeding area of the *Turdus iliacus* population on the Eurasian mainland , which during autumn almost entirely migrates towards the winter grounds in south and south-western Europe [30].

**References in Additional File**

1. Schwaiger M, Cassinotti P. Development of a quantitative real-time RT-PCR assay with internal control for the laboratory detection of tick borne encephalitis virus (TBEV) RNA. J Clin Virol. 2003;27:136–45. doi:10.1016/s1386-6532(02)00168-3.
2. Ornstein K, Barbour AG. A reverse transcriptase-polymerase chain reaction assay of *Borrelia burgdorferi* 16S rRNA for highly sensitive quantification of pathogen load in a vector. Vector Borne Zoonotic Dis. 2006;6:103–12. doi:10.1089/vbz.2006.6.103.
3. Reye AL, Hübschen JM, Sausy A, Muller CP. Prevalence and seasonality of tick-borne pathogens in questing *Ixodes ricinus* ticks from Luxembourg. Appl Environ Microbiol. 2010;76:2923–31. doi:10.1128/AEM.03061-09.
4. Fiecek B, Slivinska K, Swislocka-Cutter M, Stasiak A, Werszko J, Chmielewski T, et al. The occurrence of *Borrelia miyamotoi* in *Dermacentor reticulatus* and *Ixodes ricinus* ticks in the Chornobyl Exclusion Zone, Ukraine. Sci Rep. 2024;14:28436. doi:10.1038/s41598-024-77295-9.
5. Nowak-Chmura M, Siuda K, Wegner Z, Piksa K. Species diversity of ticks (Acari: Ixodida) on migrating birds on the Baltic Sea coast of Poland. Zool Stud. 2012;51:1411–7.
6. Olsen B, Jaenson TGT, Bergstrom S. Prevalence of *Borrelia burgdorferi* sensu lato-infected ticks on migrating birds. Appl Environ Microbiol. 1995;61:3082–7. doi:10.1128/aem.61.8.3082-3087.1995.
7. Comstedt P, Bergström S, Olsen B, Garpmo U, Marjavaara L, Mejlon H, et al. Migratory passerine birds as reservoirs of lyme borreliosis in Europe. Emerg Infect Dis. 2006;12:1087–95. doi:10.3201/eid1207.060127.
8. Kjelland V, Stuen S, Skarpaas T, Slettan A. *Borrelia burgdorferi* sensu lato in *Ixodes ricinus* ticks collected from migratory birds in Southern Norway. Acta Vet Scand. 2010;52:38. doi:10.1186/1751-0147-52-38.
9. Fabri N, Heesterbeek H, Cromsigt J, Ecke F, Sprong H, Nijhuis L, et al. Exploring the influence of host community composition on the outbreak potential of *Anaplasma phagocytophilum* and *Borrelia burgdorferi* s.l. Ticks Tick Borne Dis. 2012;14:2901–10. doi:10.1111/j.1462-2920.2012.02850.x.
10. Amore G, Tomassone L, Grego E, Ragagli C, Bertolotti L, Nebbia P, et al. *Borrelia lusitaniae* in immature *Ixodes ricinus* (Acari: Ixodidae) feeding on common wall lizards in Tuscany, central Italy. J Med Entomol. 2007;44:303–7. doi:10.1093/jmedent/44.2.303.
11. Borsan SD, Ionica AM, Galon C, Toma-Naic A, Pestean C, Sándor AD, et al. High Diversity, Prevalence, and Co-infection Rates of Tick-Borne Pathogens in Ticks and Wildlife Hosts in an Urban Area in Romania. Front Microbiol. 2021;12:645002. doi:10.3389/fmicb.2021.645002.
12. Capligina V, Salmane I, Keiss O, Vilks K, Japina K, Baumanis V, et al. Prevalence of tick-borne pathogens in ticks collected from migratory birds in Latvia. Ticks Tick Borne Dis. 2014;5:75–81. doi:10.1016/j.ttbdis.2013.08.007.
13. Chvostac M, Spitalska E, Vaclav R, Vaculova T, Minichova L, Derdakova M. Seasonal Patterns in the Prevalence and Diversity of Tick-Borne *Borrelia burgdorferi* Sensu Lato, *Anaplasma phagocytophilum* and *Rickettsia* spp. in an Urban Temperate Forest in South Western Slovakia. Int J Environ Res Public Health. 2018;15:994. doi:10.3390/ijerph15050994.
14. Franke J, Moldenhauer A, Hildebrandt A, Dorn W. Are birds reservoir hosts for *Borrelia afzelii*? Ticks Tick Borne Dis. 2010;1:109–12. doi:10.1016/j.ttbdis.2010.03.001.
15. Geller J, Nazarova L, Katargina O, Leivits A, Järvekülg L, Golovljova I. Tick-borne pathogens in ticks feeding on migratory passerines in western part of Estonia. Vector Borne Zoonotic Dis. 2013;13:443–8. doi:10.1089/vbz.2012.1192.
16. Gryczynska A, Welc-Faleciak R. Long-term study of the prevalence of *Borrelia burgdorferi* s.l. infection in ticks (*Ixodes ricinus*) feeding on blackbirds (*Turdus merula*) in NE Poland. Exp Appl Acarol. 2016;70:381–94. doi:10.1007/s10493-016-0082-x.
17. Gryczynska A, Kowalec M. Different Competence as a Lyme Borreliosis Causative Agent Reservoir Found in Two Thrush Species: The Blackbird (*Turdus merula*) and the Song Thrush (*Turdus philomelos*). Vector Borne Zoonotic Dis. 2019;19:450–2. doi:10.1089/vbz.2018.2351.
18. Hanincova K, Taragelova V, Koci J, Schafer SM, Hails R, Ullmann AJ, et al. Association of *Borrelia garinii* and *B. valaisiana* with songbirds in Slovakia. Appl Environ Microbiol. 2003;69:2825–30. doi:10.1128/AEM.69.5.2825-2830.2003.
19. Heylen D, Fonville M, Docters Van Leeuwen A, Stroo A, Duisterwinkel M, Van Wieren S, et al. Pathogen communities of songbird-derived ticks in Europe's low countries. Parasit Vectors. 2017;10:487. doi:10.1186/s13071-017-2423-y.
20. Hornok S, Csorgo T, de la Fuente J, Gyuranecz M, Privigyei C, Meli ML, et al. Synanthropic birds associated with high prevalence of tick-borne rickettsiae and with the first detection of *Rickettsia aeschlimannii* in Hungary. Vector Borne Zoonotic Dis. 2013;13:77–83. doi:10.1089/vbz.2012.1032.
21. Humair PF, Turrian N, Aeschlimann A, Gern L. *Ixodes ricinus* immatures on birds in a focus of Lyme Borreliosis. Folia Parasitol (Praha). 1993;40:237–42.
22. Kipp S, Goedecke A, Dorn W, Wilske B, Fingerle V. Role of birds in Thuringia, Germany, in the natural cycle of *Borrelia burgdorferi* sensu lato, the Lyme disease spirochaete. Int J Med Microbiol. 2006;296:125–8. doi:10.1016/j.ijmm.2006.01.042.
23. Lommano E, Dvorak C, Vallotton L, Jenni L, Gern L. Tick-borne pathogens in ticks collected from breeding and migratory birds in Switzerland. Ticks Tick Borne Dis. 2014;5:871–82. doi:10.1016/j.ttbdis.2014.07.001.
24. Mannelli A, Nebbia P, Tramuta C, Grego E, Tomassone L, Ainardi R, et al. *Borrelia burgdorferi* sensu lato infection in larval *Ixodes ricinus* (Acari: Ixodidae) feeding on blackbirds in northwestern Italy. J Med Entomol. 2005;42:168–75. doi:10.1093/jmedent/42.2.168.
25. Michalik J, Wodecka B, Skoracki M, Sikora B, Stańczak J. Prevalence of avian-associated *Borrelia burgdorferi* s.l. genospecies in *Ixodes ricinus* ticks collected from blackbirds (*Turdus merula*) and song thrushes (*T. philomelos*). Int J Med Microbiol. 2008;298:129–38. doi:10.1016/j.ijmm.2008.01.008.
26. Norte AC, Ramos JA, Gern L, Núncio MS, Lopes de Carvalho I. Birds as reservoirs for *Borrelia burgdorferi* s.l. in Western Europe: circulation of *B. turdi* and other genospecies in bird-tick cycles in Portugal. Environ Microbiol. 2013;15:386–97. doi:10.1111/j.1462-2920.2012.02850.x.
27. Norte AC, da Silva LP, Tenreiro PJQ, Felgueiras MS, Araújo PM, Lopes PB, et al. Patterns of tick infestation and their *Borrelia burgdorferi* s.l. infection in wild birds in Portugal. Ticks Tick Borne Dis. 2015;6:743–50. doi:10.1016/j.ttbdis.2015.06.009.
28. Palomar AM, Portillo A, Santibáñez P, Mazuelas D, Roncero L, Gutiérrez O, et al. Presence of *Borrelia turdi* and *Borrelia valaisiana* (Spirochaetales: Spirochaetaceae) in Ticks Removed From Birds in the North of Spain, 2009-2011. J Med Entomol. 2017;54:243–6. doi:10.1093/jme/tjw158.
29. Taragel'ová V, Koci J, Hanincova K, Kurtenbach K, Derdáková M, Ogden NH, et al. Blackbirds and song thrushes constitute a key reservoir of *Borrelia garinii*, the causative agent of Borreliosis in Central Europe. Appl Environ Microbiol. 2008;74:1289–93. doi:10.1128/AEM.01060-07.
30. del Hoyo J, Elliott A, Christie DA. Handbook of the Birds of the World. Volume 10: Cuckoo-shrikes to Thrushes. Barcelona: Lynx Edicions; 2005.
